# Cysteine/Histidine-Dependent Amidohydrolase/Peptidase (CHAP)-Displayed Nano Phages: Antimicrobial Function against Methicillin-Resistant *Staphylococcus aureus* (MRSA)

**Published:** 2020

**Authors:** Golnar Rahimzadeh, Pooria Gill, Mohammad Sadegh Rezai

**Affiliations:** 1. Pediatric Infectious Diseases Research Center, Mazandaran University of Medical Sciences, Sari, Iran; 2. Nanomedicine Group, Immunogenetics Research Center, Mazandaran University of Medical Sciences, Sari, Iran

**Keywords:** Bacteriophages, Endolysin, Methicillin-resistant *Staphylococcus aureus*

## Abstract

**Background:**

Emergence and prevalence of multi drug resistance strains such as Methicillin-Resistant *Staphylococcus aureus* (MRSA) call for new antibacterial option. Endolysins as a new option is suggested. The phage display technique is suggested for production of recombinant endolysins. The recombinant endolysins displayed nano phages specifically lysis bacteria, which penetrate to the depth of tissue and the effective dose is reduced.

**Methods:**

*CHAPK* gene was ligated in T7Select vector arms in T7Select10-3b cloning kit. To produce recombinant nano phages, ligation reaction was added directly to the packaging extract. Recombinant nano phages were amplified by Double Layer Agar assay (DLA). The recombinant nano phages were characterized using TEM. Size of recombinant nano phages was determined using DLS. The spot test was performed to confirm CHAPk -displayed on the surface of nano phages. The turbidimetry was used to investigate lytic activity of recombinant nano phages against MRSA ATCC No. 33591.

**Results:**

The results showed recombinant nano phages belonged to order Caudovirales and family Podoviridae with titer 2×10^7^ *PFU/ml*. According to the results of DLS, size of recombinant nano phages was 71 nm. Formation inhibition zone confirmed the presence of CHAPk on the surface of nano phage phenotypically. The turbidimetry showed lytic activity recombinant nano phages against MRSA after 5 *min*.

**Conclusion:**

This study suggests that CHAPk -displayed nano phages can be effective in MRSA infections.

## Introduction

With developments in treatment of infectious diseases, Multi Drug Resistance (MDR) strains such as Methicillin-Resistant *Staphylococcus aureus* (MRSA) have emerged and expanded within the last 50 years 1–3. Prevalence of MDR strains and return of infections call for new antibacterial option. Bacteriophages and their endolysins as an interesting therapeutic option have attracted the attention of researchers 4–9.

Endolysins as hydrolytic enzymes are produced by bacteriophages which destroy the cell wall of bacteria at the final stage of lytic cycle. Lysines, specifically lysis bacteria, unlike antibiotics have the following features; resistance to them is not reported. They have no effect on normal microflora and no immune response is created against them 7,10–12. The truncated recombinant endolysins destroy gram-positive bacteria exogenously. In previous studies, cloning method was used to produce recombinant lysine such as LysK, phill, Twort, 187, P68, phiWMY, SAL-1, SAP-2, ClyS, and MV-L against *Staphylococcus aureus* (*S. aureus*) strains 8,11–15. In order to produce recombinant endolysins by nano-carrier technology, the phage display is suggested. This technique eliminates cloning method and high cost enzymatic reactions 16. By phage display, the peptide or protein gene is expressed so peptide or protein is displayed on the surface of nano-carrier. Passive and active targeting are caused by nano-carrier technology. In passive targeting, the recombinant endolysin penetrates to the depth of tissue. The effective dose is reduced, and there is no toxic effect on eukaryotic cells. Active targeting specifically lysis MDR strains have no effect on microflorabacteria 8,16–18.

One of the truncated recombinant lysine is cysteine- histidine-dependent amino hydrolase/peptidase, CHAPK, 18.6 *kDa*, the part of Lys K that specifically cleaves the peptide bonds between D-alanine and glycine in the pentaglycine cross bridges of *S. aureus* cell wall. The purpose of this study was to design and manufacture recombinant nano phage by phage display 8,15,19,20. With expression of *CHAPK* gene, the recombinant CHAPk -displayed on the surface of nano- carrier. The smart recombinant nano-phages specifically lysis MRSA with the least effective dose, have high penetration and no side effects. Also, they produce on a large scale with moderate cost 8,16.

To the best of our knowledge, production of recombinant nano-bacteriophages by phage display has not been performed. This study suggests that CHAPk-displayed nano phages can be effective in MRSA infections.

## Materials and Methods

### Preparation of MRSA strain

MRSA ATCC No. 33591 strain was confirmed using conventional methods such as microscopic examination of gram stain smear, tube coagulase, catalase, DNase, and mannitol fermentation tests were performed. To detect MRSA antibiotic resistance, the Kirby–Bauer technique was carried out using Methicillin and Oxacillin antibiotic discs 5,6,8.

### Preparation of CHAPK gene

*CHAPK* gene was selected from the NCBI with sequence ID, KY974323.1, considering appropriate cutting positions by restriction enzymes, Hind III and EcoRI. This sequence with 501 *bp* was synthesized by Bioneer Company (South Korea). The *CHAPK* gene was cut in pBHA vector by double enzyme digestion. 10X Buffer Tango, 200 *μg/ml* inserted *CHAPK* gene in pBHA vector, 10 *U/μl* and EcoRI, 10 *U/μl* Hind III (Thermo scientific, U.S.A) and deionized water were combined and incubated at 37*°C* for 16 *hr*. To confirm double enzyme digestion reaction, electrophoresis was performed with TBE 1% and agarose 1%, 140 *v* for 45 *min*. Then, *CHAPK* gene was cut and extracted from gel by the EZ-10 Spin Column DNA Gel Extraction Kit (Bio Basic, Canada) 8,21–23.

### Ligation of inserts in T7Select vector

The *CHAPK* gene was ligated in T7Select vector in T7Select10-3b Cloning Kit (Novagen, USA). The molar ratios 3:1, 500 *ng/μl* T7Select vector arms were added to 184 *μg/ml CHAPK* gene. T4 DNA ligase, 10x T4 DNA ligase buffer (Takara, Japan), and deionized water were added and incubated at 16*°C* for 3 *hr* 8,21–23.

### In vitro packaging

The T7Select packaging extract in T7Select10-3b Cloning Kit (Novagen, USA) was thawed on ice. The ligation reaction was added directly per 25 *μl* packaging extract. Then, it was gently mixed by stirring with a pipette tip. The reaction was incubated at room temperature (22*°C*) for 2 *hr*. The reaction was stopped by adding 70 *μl* sterile Luria-Bertani medium. Then, 20 *μl* chloroform was added and mixed gently and the packaging reaction was stored at 4*°C* 8,21–23.

### Amplification of the packaged phages

In order to determine the titer of recombinant nano phages and their amplification, Double Layer Agar (DLA) assay was done. The BL21 as host strain in T7Select10-3b Cloning Kit (Novagen, USA) was inoculated in M9 Luria-Bertani medium and shook at 37*°C* until the OD600 reached 1. The serial dilutions of CHAPk -displayed nano phages were prepared in 7 tubes using sterile Luria-Bertani medium. Tubes 8 and 9 were negative and positive controls which consist of M9 Luria-Bertani medium and BL21, respectively. 100 *μl* of diluted CHAPk -displayed nano phages were added to 250 *μl* host cell. Then, top agarose was added to each tube and poured on plate. The plates were allowed to sit undisturbed for several minutes until the top agar was hardened. They were incubated at room temperature overnight. After that, the plaques were counted and the CHAPk -displayed nano phages titers were calculated 5,6,8.

### Confirmation of recombinant nano phages

PCR reaction was performed to approve ligation of CHAPk in T7Select vector. The plaques were removed with Pasteur pipettes and were transferred to sterile microtube. Then microtube was incubated for 10 *min* at 80*°C*. PCR reaction was performed. The sample consisted of 10 *μl* master mix, 1 *μl* up primer, 1 *μl* down primer, 4 *μl* deionize water, and 4 *μl* CHAPk in vector. The positive control consisted of 10 *μl* master mix, 1 *μl* up primer, 1 *μl* down primer, 4 *μl* deionize water, and 4 *μl CHAPK* gene in pBHA vector. The negative control consisted of 10 *μl* master mix, 1 *μl* up primer, 1 *μl* down primer, and 8 *μl* deionize water. PCR products were electrophoresed with TBE 1% and agarose 1%, 140 *v* for 45 *min* 8.

### Characterization of recombinant nano phages using TEM

The recombinant nano phages were concentrated by centrifugation at 25,000×*g* for 60 *min* followed by two washes in 0.1 *M* neutral ammonium acetate. The recombinant nano phages were deposited on carbon-coated copper grids and stained by 2% uranyl acetate (pH=4–4.5). After staining, the recombinant nano phages were observed on a Zeiss EM 900 electron microscope at 150 *Kv* 5,6,8.

### Determination of recombinant nano phages size using DLS

To determine the size of recombinant nano phages, DLS was done (ZEN3600, MALVERN). 1 *ml* of the recombinant nano phages was poured into quotes and the average size was determined 8.

### Purification of recombinant nano phages

1 *ml* of BL21 as the host strain in T7Select10-3b Cloning Kit (Novagen, U.S.A) in 200 *ml* sterile M9 Luria-Bertani was shacked at 250 *rpm* 37*°C* until the OD600 reached 0.6–0.8. The culture was infected by adding 0.02 *ml* high titer T7 phage lysate and shaking continued at 37*°C* until 1–1.5 *hr*. Lysed culture was cooled at room temperature. Recombinant DNase I (Takara, Japan) was added and incubated for 30 *min* at room temperature. 1 *M* NaCl was added and the flask was left on ice for an hour. Cell debris was removed by centrifugation at 11000×*g* for 10 *min* at 4*°C* and super-natant was collected. Solid polyethylene glycol (PEG, Sigma Ltd., UK) was added to final concentration of 10% (w/v) and slowly dissolved at room temperature; then, the flask was incubated at 4*°C* for at least 1 *hr*. The precipitated bacteriophage particles were recovered by centrifugation at 11000×*g* for 10 *min* at 4*°C* and the pellet was re-suspended in SM buffer. An equal volume of chloroform was added and culture was centrifuged at 300×*g* for 15 *min* at 4*°C*. The upper aqueous phase was removed and CHAPk -displayed nano phages were pelleted by centrifugation at 11000 × *g* for 2 *hr* at 4*°C*. The CHAPk -displayed nano phages pellets were re-suspended in 1–2 *ml* of SM buffer at 4*°C* overnight 8,24.

### Confirmation of expressed CHAPk on the surface of nano phages

Phenotypically, to confirm CHAPk -displayed nano phages, spot test was performed. An overnight culture of the MRSA (100 *μl*) was mixed in soft agar. Then, the contents were poured in petri dishes containing bottom agar. Then, 10 *μl* of the recombinant nano phage was poured on the solidified soft agar. The petri dishes were incubated at 37*°C* overnight. The following day, formation of inhibition zone was checked 5,6,8.

### Investigation of lytic activity of CHAPk -displayed nano phages

In order to investigate the lytic activity of CHAPk -displayed nano phages, the turbidimetry was used. The MRSA ATCC No. 33591 in Luria-Bertani medium was inoculated and shook at 37*°C* until the OD600 *nm* reached 0.1 (1.5×10^8^ *CFU/ml*). Then serial dilutions of the MRSA bacteria were prepared in 6 tubes using sterile Luria-Bertani medium. The tube 0 consisted of MRSA bacteria and tube 7 consisted of Luria-Bertani medium which were positive control and negative control, respectively. Then, 100 *μl* from diluted 6 tubes was transferred to sterile tubes separately. 100 *μl* CHAPk -displayed nano phages (2×10^9^ *PFU/ml*) were added to the 6 tubes. After 5 and 10 *min*, the concentration of MRSA ATCC No.33591 was detected by spectrophotometer at OD600 *nm*. Similar to the aforementioned method, natural bacteriophages (2×10^9^ *PFU/ml*) were used as positive control group. Natural bacteriophages were isolated in our previous study from sewage in Sari Bouali Sina Hospital, Mazandaran province in north of Iran^8^.

## Results

### Preparation of CHAPK gene

**Figure 1.**
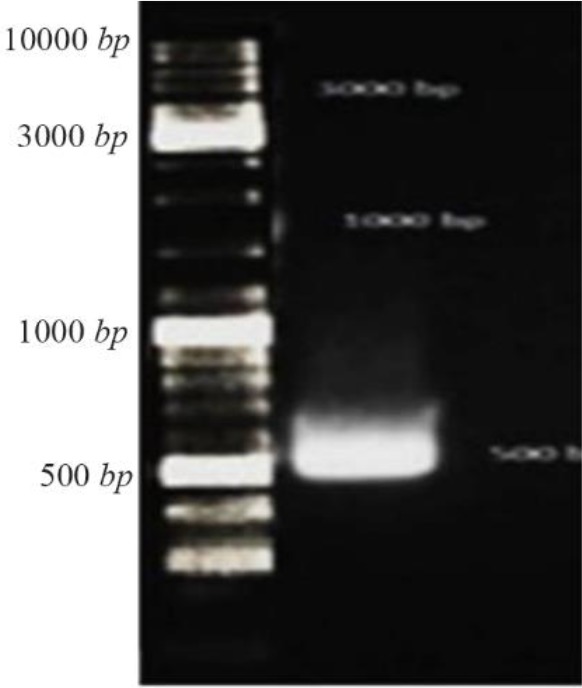
Gel electrophoresis *CHAPK* gene, *CHAPK* gene was located in 500 *bp*. Electrophoresis with TBE 1% and agarose 1%, 140 *v* for 40 *min*.

The result of electrophoresis of inserted *CHAPK* genein pBHA vector after double digestion reaction by restriction enzymes Hind III and EcoRI showed that *CHAPK* gene was located in 500 *bp* (Figure 1).

### Amplification of the packaged phages

**Figure 2.**
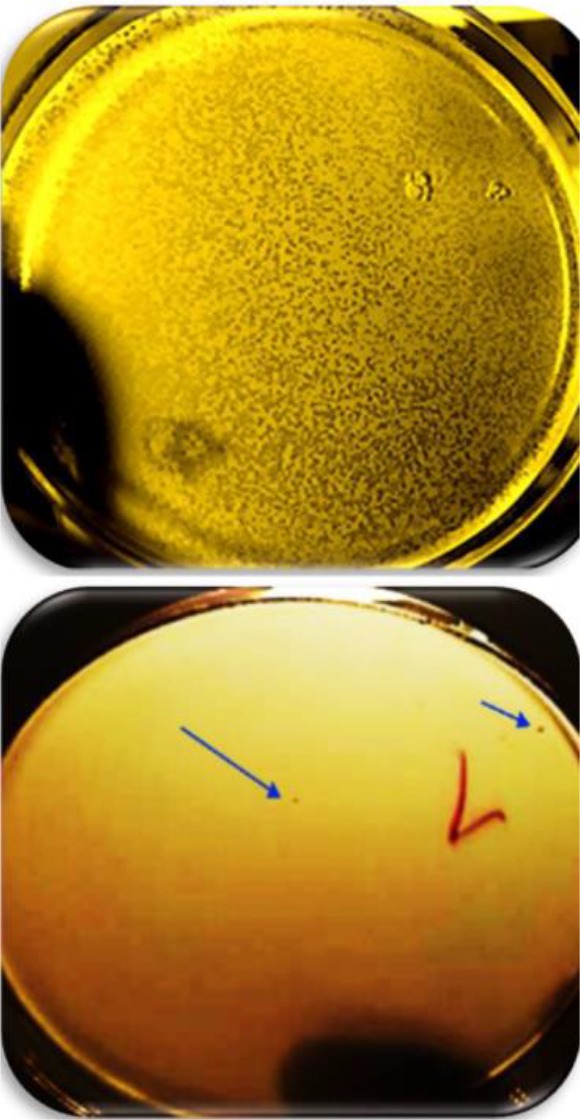
A) Titer of recombinant nano phages in the first dilution from serial dilution, B) Titer of recombinant nano phages in the seventh dilution from serial dilution was calculated 2×10^7^ *PFU/ml*.

For amplification and detection of recombinant nano phages titer, DLA assay technique was used. The number of plaques was counted after 24 *hr*. Plaque-forming units per milliliter were calculated as follows: the number of plaques×10×inverse of the dilution factor. Titer of recombinant nano phages was 2×10^7^ *PFU/ml* (Figure 2).

### Confirmation of recombinant nano phages

**Figure 3.**
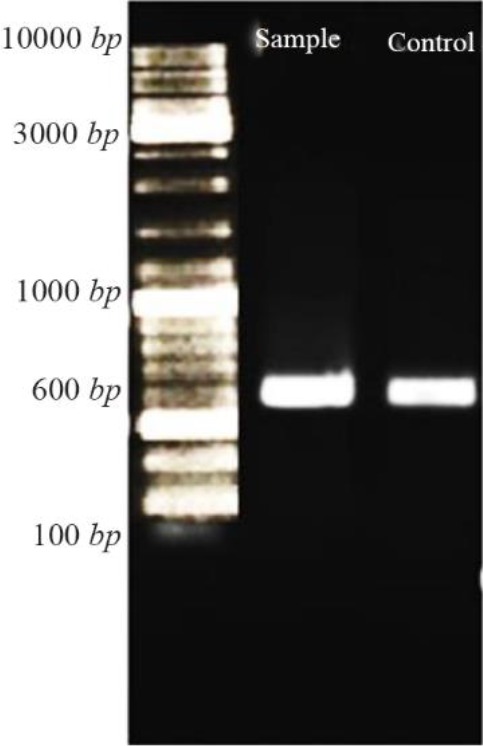
Confirmation of ligation CHAPk in T7Select vector using PCR reaction. After electrophoresis, the PCR product and positive control were located at 600 *bp*.

After ligation of *CHAPK* gene in T7Select vector, PCR reaction was performed. Then, PCR product was electrophoresed. The PCR product located at 600 *bp* (Figure 3).

### Characterization of recombinant nano phages using TEM

**Figure 4.**
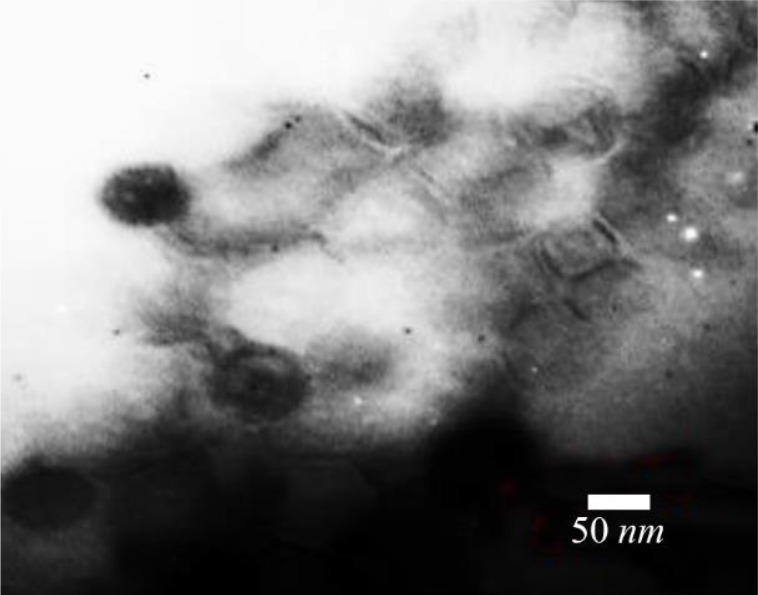
Electron micrographs showed recombinant nano phage belonged to Podoviridae family. Negatively stained with 2% uranyl acetate (pH=4–4.5). Voltage 150 *Kv*, the scale bar represents 50 *nm*.

Electron microscopy was performed by negative staining of 2% uranyl acetate (pH=4–4.5) at 150 *Kv* voltage. The results showed one recombinant nano phage group with an icosahedral head (50 *nm*) and a short non-contractile tail (28 *nm*). The recombinant nano phage group belonged to order Caudovirales and family Podoviridae (Figure 4).

### Determination of recombinant nano phages size using DLS

**Figure 5.**
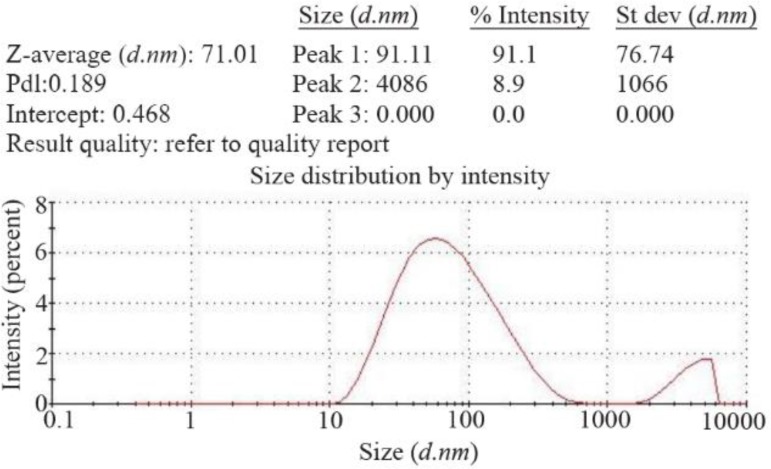
Size of recombinant nano phages detected by DLS method (ZEN3600, MALVERN). The size of recombinant nano phages was 71 *nm*.

To determine the size of recombinant nano phage, DLS technique was used by ZEN3600, MALVERN. This instrument with back scattering detector (Laser wavelength) was used for measuring the size of recombinant nano phages (diameter) in batch mode at 25*°C* in a quartz cuvette. According to the results, the size of recombinant nano phages was 71 *nm* (Figure 5).

### Confirmation of expressed CHAPk on the surface of nano phages

**Figure 6.**
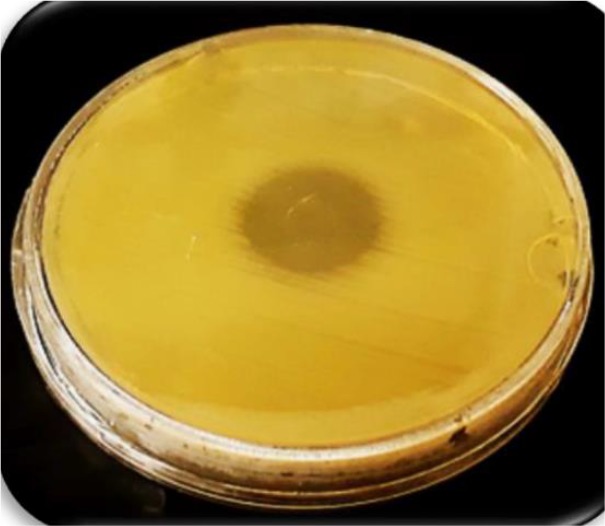
The inhibition zone in the spot test confirmed CHAPk displayed on the surface of nano phages. The recombinant nano phages had lytic activity against MRSA ATCC No. 33591.

The spot test was used to confirm CHAPk displayed on the surface of nano phages. The inhibition zone was observed. This demonstrated that the CHAPk displayed on the surface of nano phages. The recombinant nano phages had lytic activity against MRSA ATCC No. 33591 (Figure 6).

### Investigation of lytic activity of CHAPk -displayed nano phages

**Figure 7.**
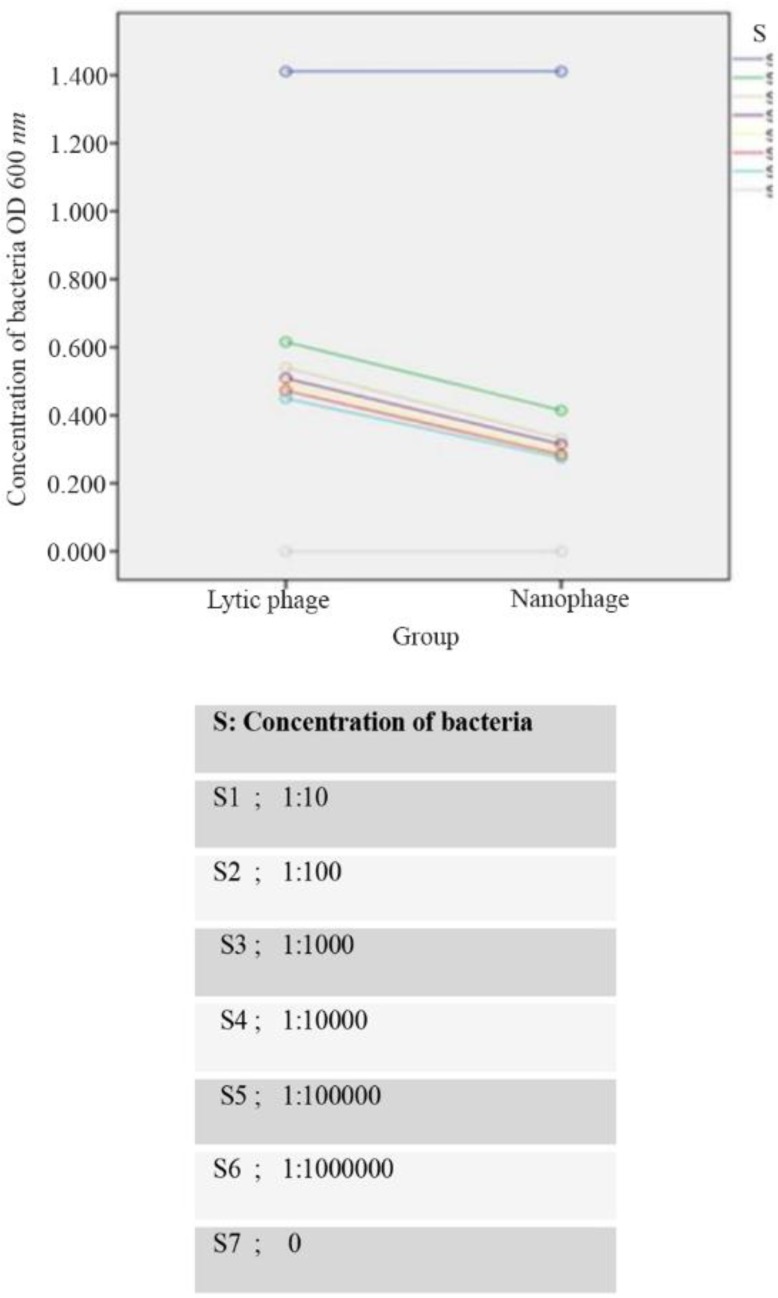
Comparison of bacterial concentration in two groups, natural phage and recombinant nano phage.

The turbidimetry was used to investigate the lytic activity of CHAPk -displayed nano phages against MRSA ATCC No. 33591. The results showed CHAPk -displayed nano phages (2×10^7^ *PFU/ml*) had lytic activity against MRSA after 5 *min* of incubation (Figure 7).

## Discussion

Currently, recombinant endolysins are suggested as a novel class of antimicrobial 7,10,11. Recombinant lysine exogenously destroys gram-positive bacteria, has no effect on normal microflora, and so far no forms of resistance to them has been identified 8,13–15.

Nano carrier technology is a safe and efficient approach for encapsulation of recombinant lysine. It creates recombinant nano phages with active and passive targeting. Manipulated recombinant nano phages with these features specifically lysis bacterial cells with the least effective dose, have high penetrating power, and no side effects 8,16,18.

In order to generate recombinant nano phages, the phage display technique is suggested 8,17,18. In this method, nano phages act as nano-carriers in which peptides or proteinsare displayed on their surface caused by expression of peptides or protein gene in a low-cost production system such as *Escherichia coli (E. coli)*. Phage display is an inexpensive technology since phages are self-replicating, thus, the recombinant peptides or proteins are produced in a large scale. In phage display technique, recombinant peptide or protein is purified by the sensitive and specific bioppaning procedure, so, there would be no problem in pharma- ceutical manipulations 8,17,18,24.

In this study, CHAPk -displayed nano phages against MRSA strains were produced by phage display technique. The *CHAPK* gene was selected from reference sequence database in the NCBI. CHAPk-displayed nano phages were fused to the C-terminal of gp10B capsomers using expression vector containing *CHAPK* gene by T7 bacteriophage. CHAPk -displayed nano phages belonged to the Podoviridae family (71 *nm*, titer of 2×10^9^ *PFU/ml*).

Some researchers produced truncated recombinant CHAPK against *S. aureus* strain Xen29 (3.7×10^3^ *CFU/ml*) and *S. aureus* strain DPC5246 (1.29×10^6^ *CFU/ml*) by cloning method with pQE60 expression vector and overexpression in *E. coli* XL1-Blue host cell. It was then purified by ion-exchange chromatography. 31.25 *μg/ml* CHAPK was incubated with 200 *μl S. aureus* DPC5246 (1.29×10^6^ *CFU/ml*) in TSBg at 37°*C* for 24 *hr*, so, it completely prevented the formation of *S. aureus* biofilms. CHAPK can be used as a spray for elimination of *S. aureus* from the surface of mammalian skin in 30 *min*. Fenton *et al* reported that 10 *μg/ml* of CHAPK reduced 108 (*CFU/ml*) *S. aureus* by 4-log in 15 *min* at pH=9 19,20,25,26.

But, in the current study, CHAPk-displayed nano phages were generated by phage display technique with expression of *CHAPK* gene on the surface of T7 phages. Efficiency of phage display technique is more than that of the cloning method because phages are self-replicating, so, the highest amount of recombinant proteins is produced in minimum time. In our study, CHAPk -displayed nano phages (2×10^9^ *PFU/ml*) were incubated at 37*°C* in Luria-Bertani broth. Recombinant nano phage showed lytic activity effect against MRSA ATCC No. 33591(1.5×10^8^ *CFU/ml*). After 5 *min*, due to its nano dimensions and its greater permeability, lytic phage showed lytic effect against MRSA (1.5×10^8^ *CFU/ml*) after 20 *min*.

In our study, according to pI=6.7 of CHAPK and gp10B capsomers (achieved from http://www.expasy.org), lytic activity of CHAPk -displayed nano phages was assayed at pH=6.7–7. In some studies, the specific activities of LysK and CHAPK were assayed. CHAPK enhances lytic activity by at least twofold. The CHAPk domain (165 amino acid) is more active against live *S. aureus* than LysK (495 amino acid).In turbidity reduction assays, 10 *μg/ml* of CHAPK reduced 10^8^ CFU/*ml S. aureus* by 4-log in 15 *min* 20. But, in our study with turbidity assay, 2×10^9^ *PFU/ml* of CHAPk -displayed nano phages reduced 1.5×10^8^ *CFU/ml* of MRSA ATCC No.33591 by 5-log in 5 *min*.

Donovan *et al* found that the activity of CHAPK was threefold higher than that of the native enzyme K and amidase domain. Also, lytic activity CHAPK in addition to MRSA bacteria was reported on negative coagulase staphas such as *S. warneri, S. simulans*, and *S. epidermiditis*. The CHAPk domain alone can exhibit activity against live Staphylococcibut other *S. aureus* phage endolysins such as PlyTW and Ply187 lysis exhibit activity only in heat-killed *S. aureus* 14,27. In the present study, for confirmation of expressed CHAPk on the surface of nano phages, methods such as SDS-Page and ELISA cannot be used due to limited funding.

## Conclusion

To the best of our knowledge, in this study, phage display technique was used in nano medicine to design and produce CHAP_k_ -displayed nano phages against MRSA ATCC No. 33591 *in vitro*. Antibacterial effects of CHAPk -displayed nano phages are higher than truncated recombinant CHAPk against MRSA strain. CHAPk -displayed nano phages reduced the effective dose and lytic time.
